# Differential Impact of Nevirapine on Artemether-Lumefantrine Pharmacokinetics in Individuals Stratified by *CYP2B6* c.516G>T Genotypes

**DOI:** 10.1128/AAC.00947-19

**Published:** 2020-02-21

**Authors:** Sa’ad T. Abdullahi, Julius O. Soyinka, Adeniyi Olagunju, Rahman A. Bolarinwa, Olusola J. Olarewaju, Moji T. Bakare-Odunola, Markus Winterberg, Joel Tarning, Andrew Owen, Saye Khoo

**Affiliations:** a Department of Pharmaceutical Chemistry, Obafemi Awolowo University, Ile-Ife, Nigeria; b Department of Pharmaceutical and Medicinal Chemistry, University of Ilorin, Ilorin, Nigeria; c Department of Molecular and Clinical Pharmacology, University of Liverpool, Liverpool, United Kingdom; d Department of Haematology, Obafemi Awolowo University Teaching Hospitals Complex, Ile-Ife, Nigeria; e Mahidol-Oxford Tropical Medicine Research Unit, Faculty of Tropical Medicine, Mahidol University, Bangkok, Thailand; f Centre for Tropical Medicine and Global Health, University of Oxford, Oxford, United Kingdom

**Keywords:** artemether-lumefantrine, nevirapine, pharmacokinetics, genetic polymorphisms, CYP2B6, malaria, HIV, human immunodeficiency virus

## Abstract

There is an increased recognition of the need to identify and quantify the impact of genetic polymorphisms on drug-drug interactions. This study investigated the pharmacogenetics of the pharmacokinetic drug-drug interaction between nevirapine and artemether-lumefantrine in HIV-positive and HIV-negative adult Nigerian subjects. Thirty each of HIV-infected patients on nevirapine-based antiretroviral therapy and HIV-negative volunteers without clinical malaria, but with predetermined *CYP2B6* c.516GG and TT genotypes, were administered a complete treatment dose of 3 days of artemether-lumefantrine. Rich pharmacokinetic sampling prior to and following the last dose was conducted, and the plasma concentrations of artemether/dihydroartemisinin and lumefantrine/desbutyl-lumefantrine were quantified using tandem mass spectrometry. Pharmacokinetic parameters of artemether-lumefantrine and its metabolites in HIV-infected patients on nevirapine were compared to those in the absence of nevirapine in HIV-negative volunteers. Overall, nevirapine reduced exposure to artemether and desbutyl-lumefantrine by 39 and 34%, respectively. These reductions were significantly greater in GG versus TT subjects for artemether (ratio of geometric mean [90% confidence interval]: 0.42 [0.29 to 0.61] versus 0.81 [0.51 to 1.28]) and for desbutyl-lumefantrine (0.56 [0.43 to 0.74] versus 0.75 [0.56 to 1.00]). On the contrary, it increased exposure to dihydroartemisinin and lumefantrine by 47 and 30%, respectively. These increases were significantly higher in TT versus GG subjects for dihydroartemisinin (1.67 [1.20 to 2.34] versus 1.25 [0.88 to 1.78]) and for lumefantrine (1.51 [1.20 to 1.90] versus 1.08 [0.82 to 1.42]). This study underscores the importance of incorporating pharmacogenetics into all drug-drug interaction studies with potential for genetic polymorphisms to influence drug disposition.

## Abstract (precis)

There is an increased recognition of the need to identify and quantify the impact of genetic polymorphisms on drug-drug interactions. This study investigated the pharmacogenetics of the pharmacokinetic drug-drug interaction between nevirapine and artemether-lumefantrine in HIV-positive and HIV-negative adult Nigerian subjects.

## TEXT

HIV and malaria are endemic diseases in many developing countries (1). The distribution of both diseases overlaps in many regions of the world, particularly in sub-Saharan Africa (2). Management of coinfection is a major challenge to public health most especially in low-resource settings (3). Treatment for HIV in these populations is often limited by cost, and the relatively inexpensive nevirapine-based antiretroviral therapy (ART) regimens are still used as alternative first-line regimens (4, 5). Artemether-lumefantrine is a highly effective fixed-dose artemisinin-based combination therapy, and the most widely used of the World Health Organization recommended first-line treatments for uncomplicated Plasmodium falciparum malaria (6).

While artemether is primarily metabolized by CYP3A4/5 and 2B6 to the biologically active main metabolite dihydroartemisinin, which is further converted to inactive metabolites through UDP-glucuronosyltransferases catalyzed glucuronidation by UGT1A9 and UGT2B7 with minor contribution from UGT1A1 and UGT1A8 (7–9). Lumefantrine is primarily N-debutylated to desbutyl-lumefantrine by CYP3A4/5 (10). In addition, nevirapine is a metabolic substrate and also a known inducer of CYP3A4 and CYP2B6 (11, 12). This creates a potential for important drug-drug interactions following coadministration of artemether-lumefantrine to HIV-infected patients on nevirapine-based ART regimens who are being treated for recurrent malaria (13). Moreover, *CYP2B6* is one of the most polymorphic CYP genes in humans and the *CYP2B6* c.516G>T (in allele CYP2B6*6) mutation, one of the most common single nucleotide polymorphisms (SNPs), has been reported to influence exposure to both nevirapine and artemether (14, 15).

Interindividual variation in the expression and activity of drug-metabolizing enzymes and transporters may be attributed to genetic and/or environmental factors and may affect drug disposition (16). Genetic polymorphisms exhibit both interindividual and interpopulation differences (17). Study of genetic variation of *CYP2B6* within and between population groups has indicated that approximately 90% of the genetic variation was due to interindividual differences, while the remaining 10% was attributed to interpopulation differences (18). Africans are known to exhibit a higher degree of this genetic variation compared to other populations (19). Minor *CYP2B6* c.516T allele frequency as high as 50% has been reported in Ghanaian, Mozambican, and Zimbabwean populations compared to almost 20 and 30% in Asian and European populations, respectively (20–23). Furthermore, frequencies of 23 to 30% for Cape Mixed Ancestry in South African, 31% in Malawian, 29 to 36% in Ugandan, 42% in Tanzanian, and 36 to 40% in Nigerian populations have been reported (23–28), hence the need to quantify the impact of this SNP on pharmacokinetic drug-drug interactions involving CYP2B6 substrates.

In addition, data on the pharmacokinetic drug interactions between nevirapine and artemether-lumefantrine in HIV-infected patients have been inconsistent and conflicting, especially with respect to lumefantrine exposure (29–34). While South African (29), Nigerian (32), and Tanzanian (33) studies and, recently, a Malawian-Ugandan (34) study reported increased lumefantrine exposures, a Ugandan study (30) and another Nigerian study (31) reported decreased exposures. Moreover, data on the impact of genetic polymorphisms on the drug-drug interactions between artemether-lumefantrine and nevirapine are sparse (35). We recently reported the influence of pharmacogenetic variations on the interaction of artemether-lumefantrine with nevirapine in a cohort of HIV-infected patients (36). In view of the conflicting reports and the increasing relevance of genetic polymorphisms to impact drug disposition, this study investigated influence of homozygous *CYP2B6* c.516G>T genotypes on the pharmacokinetic interaction of nevirapine with artemether-lumefantrine in HIV-infected Nigerian patients using HIV-negative volunteers as a control.

## RESULTS

### Baseline characteristics of sampled participants.

The mean (standard deviation) of age (39.8 years [9.7]), body mass index (23.6 kg^−2^ [4.9]) and genotype frequencies (%) (GG [38.7] and TT [11.3]) of the cohort of HIV-infected patients previously described (28) were not significantly different from the 30.6 years (11.8); 23.1 kg^−2^ (4.6), and GG (42.0) and TT (16.0), respectively, of HIV-negative subjects. However, the sex distribution (number of male [%]) was significantly different between the HIV-infected patients (42 [28.0%]) and HIV-negative subjects (94 [62.7%]) (*P* < 0.05). Furthermore, the mean CD4 cell count of patients with GG genotype (327 cells mm^−3^ [207]) was not significantly different from those with TT genotype (343 cells mm^−3^ [152]).

### Influence of nevirapine on artemether pharmacokinetics in pooled analysis and *CYP2B6* c.516G>T stratified cohort.

One HIV-negative and two HIV-infected subjects with poor artemether-lumefantrine dosage and blood sampling compliance were excluded from the final analysis. There was a systematic difference (imbalance) in the number of lower limit of quantitation (LLOQ) samples between groups due to the drug-drug interactions. Approximately 36.8% (i.e., 178) of the 484 artemether samples were quantified to be below the assay LLOQ, most of which, i.e., 63.5% (113), were from patients on nevirapine-based ART regimens. About 30.1% (34) of the 113 LLOQs were for the 6- and 8-h time points compared to the 76.1% (86 LLOQs) for the other time points, excluding the zero-hour (predose) time point. On the other hand, approximately 25.2% (122) of 484 of the dihydroartemisinin samples were quantified to be below the LLOQ, of which 52.5% (64) were from patients on nevirapine-based regimens. Almost 28.1% (18) of the 64 LLOQs were for the 6- and 8-h time points versus the 64.1% (41 LLOQs) for the other time points (excluding the predose LLOQs). To avoid potential bias from this unbalanced data censoring between groups, the first individual LLOQ sample in the terminal elimination phase was replaced with LLOQ/2, and the rest of the LLOQ data were omitted.

In pooled analysis, comparison of the pharmacokinetic properties of artemether with nevirapine versus without nevirapine coadministration showed that nevirapine reduced the total exposure (AUC_0-∞_) to artemether by 39% (0.43 to 0.86) in all the patients due to higher elimination (CL/F was 64% [1.17 to 2.31] greater), as shown in Table 1. On the contrary, coadministration of nevirapine resulted in a 47% (1.16 to 1.86) higher exposure to dihydroartemisinin due to 32% (0.54 to 0.86) lower CL/F. Nevirapine also resulted in an average 58% (0.31 to 0.56) reduction in the metabolic ratio of artemether to dihydroartemisinin.

**TABLE 1:** Effect of nevirapine on artemether and dihydroartemisinin pharmacokinetics in all subjects^*a*^

Pharmacokinetic parameter	Artemether-lumefantrine alone (*n* = 22)	Artemether-lumefantrine with nevirapine (*n* = 18)	RoGM (90% CI)^*b*^	*P*^*c*^
Artemether				
*C*_max_ (ng/ml)	34.0 (14.8)	21.8 (32.0)	0.64 (0.44–0.94)	0.059
*T*_max_ (h)	2.00 (1.00, 2.00)	2.00 (1.00, 2.00)	0.98 (0.73–1.31)	0.893
AUC_0–8_ (ng ⋅ h/ml)	84.4 (45.3)	50.0 (82.4)	0.59 (0.42–0.83)	**0.013**
AUC_0–∞_ (ng ⋅ h/ml)	88.6 (48.6)	54.0 (89.6)	0.61 (0.43–0.86)	**0.019**
*t*_1/2_ (h)	1.48 (0.566)	1.90 (0.824)	1.28 (1.05–1.57)	**0.044**
CL/F (liters/h)	903 (505)	1,480 (1,280)	1.64 (1.17–2.31)	**0.019**
				
Dihydroartemisinin				
*C*_max_ (ng/ml)	53.4 (25.7)	86.0 (42.7)	1.61 (1.26–2.06)	**0.002**
*T*_max_ (h)	2.00 (1.25, 2.75)	2.00 (1.00, 2.00)	0.80 (0.59–1.08)	0.217
AUC_0–8_ (ng ⋅ h/ml)	135 (70.2)	197 (75.9)	1.46 (1.15–1.83)	**0.009**
AUC_0–∞_ (ng ⋅ h/ml)	140 (73.7)	205 (78.2)	1.47 (1.16–1.86)	**0.009**
*t*_1/2_ (h)	1.23 (0.574)	1.46 (0.502)	1.19 (0.99–1.42)	0.122
CL/F (liters/h)	546 (301)	371 (148)	0.68 (0.54–0.86)	**0.009**
				
Metabolic ratio^*d*^	0.634 (0.386)	0.263 (0.249)	0.42 (0.31–0.56)	**<0.001**

a All parameters are presented as geometric mean (standard deviation) except *T*_max_, which is presented as the median (interquartile range). Differences in parameters were assessed by ratio of geometric means (RoGM) and 90% confidence interval (CI). Abbreviations: *n*, sample size; *C*_max_, maximum concentration; *T*_max_, time to reach *C*_max_; AUC_0–8_, area under the concentration time curve from 0 to 8 h; AUC_0–∞_, AUC curve extrapolated to infinity; *t*_1/2_, terminal phase half-life; CL/F, oral clearance.

b Artemether-lumefantrine with nevirapine/Artemether-lumefantrine alone.

c Values in boldface are significant.

d Metabolic ratio of parent drug to metabolite.

**FIG 1:**
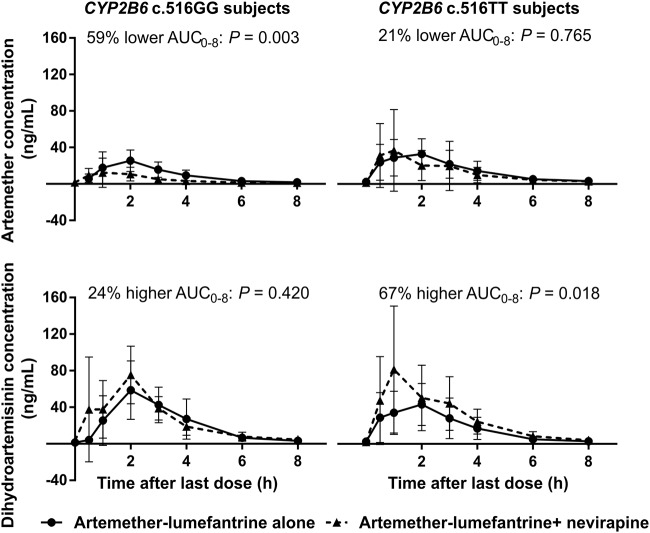
Mean (standard deviation) plasma concentration-time profiles of artemether and dihydroartemisinin in *CYP2B6* c.516GG versus TT subjects when artemether-lumefantrine was administered alone versus in the presence of nevirapine.

When stratified to *CYP2B6* c.516GG patients only, nevirapine reduced total exposure to artemether by 58% (0.29 to 0.61) due to the 137% (1.63 to 3.43) higher CL/F (Table 2). On the contrary, exposure to dihydroartemisinin was not significantly different with or without nevirapine. Coadministration of nevirapine also resulted in an average 66% (0.24 to 0.48) reduction in the metabolic ratio of artemether to dihydroartemisinin. Stratification based on *CYP2B6* c.516TT patients only resulted in a nonsignificant 19% (0.51 to 1.28) lower exposure to artemether (Table 2). However, exposure to dihydroartemisinin was significantly higher by 67% (1.20 to 2.34) due to the 40% (0.43 to 0.83) lower CL/F. Coadministration of nevirapine also resulted in an average 51% (0.35 to 0.68) lower artemether-to-dihydroartemisinin metabolic ratio. Besides, *CYP2B6* c.516TT subjects showed a 160% (1.55 to 4.37) higher exposure to artemether and a corresponding approximately 150% (1.69 to 3.70) higher artemether-to-dihydroartemisinin metabolic ratio compared to GG subjects in the presence of nevirapine. Figure 1 summarizes the influence of nevirapine on artemether and dihydroartemisinin pharmacokinetics in GG versus TT subjects, while a detailed comparison of the pharmacokinetic parameters alone with in the presence of nevirapine is presented in Table S1 in the supplemental material.

**TABLE 2:** Comparison of effect of nevirapine on artemether and dihydroartemisinin pharmacokinetics in *CYP2B6* c.516GG versus TT subjects^*a*^

Pharmacokinetic parameter	RoGM (90% CI)^*b*^		*CYP2B6* c.516GG vs TT^*c*^	
	*CYP2B6* c.516GG	*CYP2B6* c.516TT	RoGM (90% CI)	*P*
Artemether				
*C*_max_ (ng/ml)	0.45 (0.27–0.74)	0.85 (0.57–1.27)	2.84 (1.58–5.11)	**0.007**
*T*_max_ (h)	0.89 (0.57–1.38)	1.06 (0.68–1.66)	0.93 (0.56–1.53)	0.798
AUC_0–8_ (ng ⋅ h/ml)	0.41 (0.28–0.60)	0.79 (0.50–1.24)	2.59 (1.54–4.35)	**0.006**
AUC_0–∞_ (ng ⋅ h/ml)	0.42 (0.29–0.61)	0.81 (0.51–1.28)	2.60 (1.55–4.37)	**0.005**
*t*_1/2_ (h)	1.06 (0.80–1.40)	1.50 (1.12–2.01)	1.28 (0.94–1.73)	0.940
CL/F (liters/h)	2.37 (1.63–3.43)	1.23 (0.78–1.94)	0.38 (0.23–0.64)	**0.005**
				
Dihydroartemisinin				
*C*_max_ (ng/ml)	1.42 (0.94–2.16)	1.78 (1.29–2.45)	1.13 (0.78–1.66)	0.573
*T*_max_ (h)	0.67 (0.43–1.03)	0.92 (0.59–1.45)	1.18 (0.75–1.87)	0.530
AUC_0–8_ (ng ⋅ h/ml)	1.24 (0.88–1.74)	1.67 (1.20–2.31)	1.04 (0.77–1.42)	0.824
AUC_0–∞_ (ng ⋅ h/ml)	1.25 (0.88–1.78)	1.67 (1.20–2.34)	1.04 (0.77–1.42)	0.819
*t*_1/2_ (h)	1.18 (0.95–1.47)	1.19 (0.88–1.60)	1.08 (0.81–1.42)	0.654
CL/F (liters/h)	0.80 (0.56–1.13)	0.60 (0.43–0.83)	0.96 (0.70–1.31)	0.820
				
Metabolic ratio^*d*^	0.34 (0.24–0.48)	0.49 (0.35–0.68)	2.50 (1.69–3.70)	**0.001**

a Differences in parameters were assessed by ratio of geometric means (RoGM) and 90% confidence interval (CI). Subject number (*n*) = 10 in the artemether and dihydroartemisinin alone and 8 in the presence of nevirapine subgroups in the GG and 12 and 10, respectively, in the TT genotype groups. *C*_max_, maximum concentration; *T*_max_, time to reach *C*_max_; AUC_0–8_, area under the concentration time curve from 0 to 8 h; AUC_0–∞_, AUC curve extrapolated to infinity; *t*_1/2_, terminal phase half-life; CL/F, oral clearance.

b Artemether-lumefantrine plus nevirapine/artemether-lumefantrine alone.

c TT/GG (for artemether-lumefantrine plus nevirapine). Values in boldface are significant.

d Metabolic ratio of parent drug to metabolite.

### Influence of nevirapine on lumefantrine pharmacokinetics in pooled analysis and *CYP2B6* c.516G>T stratified cohort.

Overall, nevirapine coadministration enhanced total exposure to lumefantrine by 30% (1.08 to 1.55) due to the 23% (0.64 to 0.92) drop in CL/F as presented in Table 3. However, exposure to desbutyl-lumefantrine was reduced by 34% (0.54 to 0.80) in all the patients due to the 52% (1.26 to 1.85) greater CL/F. Coadministration of nevirapine, also resulted in an average 98% (1.63 to 2.39) higher lumefantrine-to-desbutyl-lumefantrine metabolic ratio.

**TABLE 3:** Effect of nevirapine on lumefantrine and desbutyl-lumefantrine pharmacokinetic parameters in all the subjects^*a*^

Pharmacokinetic parameter	Artemether-lumefantrine alone (*n* = 29)	Artemether-lumefantrine with nevirapine (*n* = 24)	RoGM (90% CI)^*b*^	*P*^*c*^
Lumefantrine				
*C*_max_ (ng/ml)	10,500 (3,210)	14,600 (5,370)	1.39 (1.19–1.62)	**0.001**
*T*_max_ (h)	4.00 (4.00, 6.00)	4.00 (1.00, 6.00)	0.74 (0.53–1.03)	0.130
AUC_0–336_ (ng ⋅ h/ml)	407,000 (127,000)	546,000 (276,000)	1.34 (1.13–1.59)	**0.006**
AUC_0–∞_ (ng ⋅ h/ml)	441,000 (138,000)	573,000 (292,000)	1.30 (1.08–1.55)	**0.015**
*t*_1/2_ (h)	106 (25.3)	83.2 (21.7)	0.79 (0.70–0.88)	**0.001**
CL/F (liters/h)	1.09 (0.360)	0.838 (0.422)	0.77 (0.64–0.92)	**0.014**
*C*_day 6_ (ng/ml)	1,210 (401)	1,660 (888)	1.37 (1.14–1.65)	**0.007**
*C*_day 10_ (ng/ml)	523 (159)	564 (338)	1.08 (0.88–1.32)	0.268
				
Desbutyl-lumefantrine				
*C*_max_ (ng/ml)	94.6 (137)	57.5 (22.0)	0.61 (0.47–0.78)	**0.002**
*T*_max_ (h)	8.00 (4.00, 8.00)	8.00 (6.00, 8.00)	1.43 (1.05–1.94)	0.056
AUC_0–336_ (ng ⋅ h/ml)	9,920 (6,040)	6,640 (2,450)	0.67 (0.55–0.81)	**0.001**
AUC_0–∞_ (ng ⋅ h/ml)	12,000 (7,140)	7,900 (2,900)	0.66 (0.54–0.80)	**0.001**
*t*_1/2_ (h)	134 (35.8)	124 (26.3)	0.92 (0.83–1.03)	0.239
CL/F (liters/h)	35.7 (17.7)	54.3 (20.8)	1.52 (1.26–1.85)	**0.001**
*C*_day 6_ (ng/ml)	38.6 (21.8)	28.4 (10.9)	0.74 (0.60–0.90)	**0.012**
*C*_day 10_ (ng/ml)	21.5 (11.5)	14.8 (6.85)	0.69 (0.56–0.84)	**0.003**
				
Metabolic ratio^*d*^	36.7 (16.1)	72.5 (29.2)	1.98 (1.63–2.39)	**<0.001**

a All parameters are presented as geometric mean (standard deviation) except for the *T*_max_ median (interquartile range). Differences in parameters were assessed by ratios of geometric means (RoGM) and 90% confidence interval (CI). Abbreviations: *n*, sample size; *C*_max_, maximum concentration; *T*_max_, time to reach *C*_max_; AUC_0–336_, area under the concentration time curve from 0 to 336 h; AUC_0–∞_, AUC curve extrapolated to infinity; *t*_1/2_, terminal phase half-life; CL/F, oral clearance; day 6 (*C*_day 6_) and day 10 (*C*_day 10_) plasma concentrations.

b Artemether-lumefantrine plus nevirapine/artemether-lumefantrine alone.

c Values in boldface are significant.

d Metabolic ratio of parent drug to metabolite.

**FIG 2:**
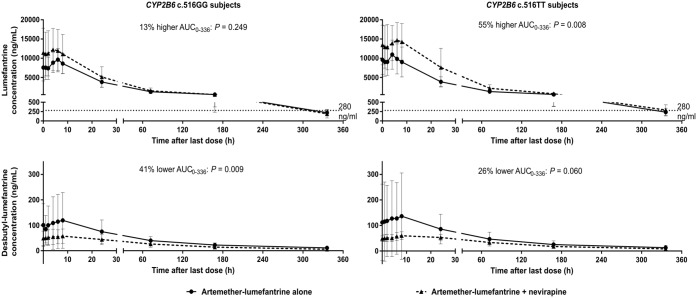
Mean (standard deviation) plasma concentration-time profiles of lumefantrine and desbutyl-lumefantrine in *CYP2B6* c.516GG versus TT subjects when artemether-lumefantrine was administered alone versus in the presence of nevirapine.

When stratified to *CYP2B6* c.516GG patients alone, nevirapine coadministration resulted in a 30% (1.04 to 1.63) higher peak levels of lumefantrine but, surprisingly, no altered total exposure (Table 4). On the contrary, exposure to desbutyl-lumefantrine was reduced by 44% (0.43 to 0.74) due to 78% (1.35 to 2.35) higher elimination. Nevirapine also resulted in an average 92% (1.42 to 2.61) higher lumefantrine-to-desbutyl-lumefantrine metabolic ratio. On stratification to *CYP2B6* c.516TT patients only, nevirapine culminated in a 51% (1.20 to 1.90) higher exposure to lumefantrine due to a 34% (0.53 to 0.84) lower CL/F (Table 4). On the contrary, total exposure to desbutyl-lumefantrine was not significantly different. Coadministration of nevirapine also resulted in an average 101% (1.55 to 2.60) rise in the metabolic ratio of lumefantrine to desbutyl-lumefantrine. Total exposures to lumefantrine and desbutyl-lumefantrine of *CYP2B6* c.516TT versus GG subjects were 46 and 32%, respectively, greater just as day 6 plasma lumefantrine concentration (*C*_day 6_) was also 49% higher. Figure 2 summarizes impact of nevirapine on lumefantrine and desbutyl-lumefantrine pharmacokinetics in GG versus TT subjects, while Table S2 provides a detailed comparison of the pharmacokinetic parameters alone with in the presence of nevirapine.

**TABLE 4:** Comparison of effect of nevirapine on lumefantrine and desbutyl-lumefantrine pharmacokinetics in *CYP2B6* c.516GG versus TT subjects^*a*^

Pharmacokinetic parameter	RoGM (90% CI)^*b*^		*CYP2B6* c.516GG vs TT^*c*^	
	*CYP2B6* c.516GG	*CYP2B6* c.516TT	RoGM (90% CI)	*P*
Lumefantrine				
*C*_max_ (ng/ml)	1.30 (1.04–1.63)	1.46 (1.18–1.81)	1.23 (0.95–1.59)	0.175
*T*_max_ (h)	0.64 (0.41–1.00)	0.86 (0.51–1.44)	1.07 (0.55–2.09)	0.862
AUC_0–336_ (ng ⋅ h/ml)	1.13 (0.88–1.44)	1.55 (1.23–1.94)	1.45 (1.09–1.92)	**0.036**
AUC_0–∞_ (ng ⋅ h/ml)	1.08 (0.82–1.42)	1.51 (1.20–1.90)	1.46 (1.10–1.95)	**0.033**
*t*_1/2_ (h)	0.73 (0.59–0.88)	0.84 (0.74–0.96)	1.20 (0.99–1.45)	0.113
CL/F (liters/h)	0.92 (0.70–1.21)	0.66 (0.53–0.84)	0.69 (0.51–0.91)	**0.034**
*C*_day 6_ (ng/ml)	1.12 (0.83–1.50)	1.65 (1.28–2.12)	1.49 (1.09–2.04)	**0.040**
*C*_day 10_ (ng/ml)	0.89 (0.67–1.18)	1.26 (0.97–1.64)	1.48 (1.06–2.07)	0.055
				
Desbutyl-lumefantrine				
*C*_max_ (ng/ml)	0.57 (0.38–0.87)	0.64 (0.44–0.93)	1.07 (0.84–1.37)	0.616
*T*_max_ (h)	1.20 (0.78–1.86)	1.65 (1.09–2.51)	1.71 (1.19–2.47)	**0.019**
AUC_0–336_ (ng ⋅ h/ml)	0.59 (0.45–0.78)	0.74 (0.55–0.99)	1.31 (1.05–1.62)	**0.047**
AUC_0–∞_ (ng ⋅ h/ml)	0.56 (0.43–0.74)	0.75 (0.56–1.00)	1.32 (1.05–1.65)	**0.049**
*t*_1/2_ (h)	0.83 (0.68–1.00)	1.03 (0.91–1.17)	1.07 (0.93–1.23)	0.441
CL/F (liters/h)	1.78 (1.35–2.35)	1.33 (1.00–1.78)	0.76 (0.61–0.95)	**0.049**
*C*_day 6_ (ng/ml)	0.66 (0.51–0.87)	0.81 (0.61–1.07)	1.33 (1.05–1.69)	0.050
*C*_day 10_ (ng/ml)	0.61 (0.45–0.81)	0.76 (0.57–1.01)	1.31 (0.98–1.75)	0.121
				
Metabolic ratio^*d*^	1.92 (1.42–2.61)	2.01 (1.55–2.60)	1.11 (0.85–1.45)	0.507

a Differences in parameters were assessed by ratios of geometric means (RoGM) and 90% confidence intervals (CI).

b Artemether-lumefantrine plus nevirapine/artemether-lumefantrine alone.

c TT/GG (for artemether-lumefantrine plus nevirapine). Subject number (*n*) = 15 in the lumefantrine and desbutyl-lumefantrine alone and 11 in the presence of nevirapine subgroups in the GG and 14 and 13 in the TT genotype groups, respectively. *C*_max_, maximum concentration; *T*_max_, time to reach *C*_max_; AUC_0–336_, area under the concentration time curve from 0 to 336 h; AUC_0–∞_, AUC curve extrapolated to infinity; *t*_1/2_, terminal phase half-life; CL/F, oral clearance; day 6 (*C*_day 6_) and day-10 (*C*_day 10_) plasma concentrations. Values in boldface are significant.

d Metabolic ratio of parent drug to metabolite.

## DISCUSSION

**FIG 3:**
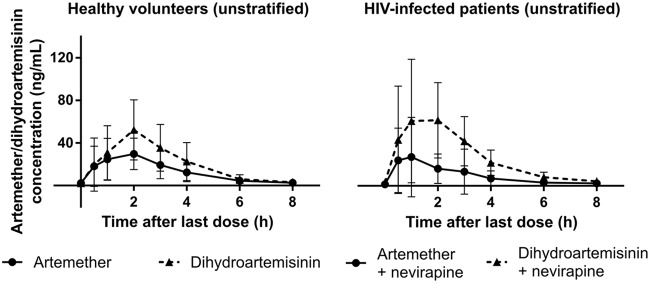
Mean (standard deviation) plasma artemether versus dihydroartemisinin concentration-time profiles of all the HIV-negative volunteers (in the absence of nevirapine) and all the HIV-infected patients (in the presence of nevirapine).

The present study showed that while total exposures to artemether and desbutyl-lumefantrine in *CYP2B6* c.516GG patients on nevirapine were significantly lower, exposures to dihydroartemisinin and lumefantrine in TT patients were higher. Similarly, metabolic ratios, which are measures of the metabolic activities of CYP2B6, were modified by genotype. While the induction of artemether metabolism (as judged by the lower artemether-to-dihydroartemisinin metabolic ratios) was blunted in TT compared to GG subjects, inhibition of lumefantrine metabolism (as demonstrated by the higher lumefantrine-to-desbutyl-lumefantrine metabolic ratios) was enhanced. The lower total exposure to artemether obtained when not stratified according to genotype agrees with results from previous studies that also reported reductions (29–31). The present study found 39% lower exposure compared to the 55, 72, and 68% reductions reported in the South African, Ugandan, and Nigerian studies, respectively (29–31). However, the observed 47% higher total exposure to dihydroartemisinin is not in agreement with the 25 and 37% reductions reported in the South African (29) and Ugandan (30) studies, respectively. It also differs from a similar parallel design study in Nigerian patients (31), as well as a study in Malawian and Ugandan pediatric patients (34) that found 23 and 22% reductions, respectively. The present findings, however, agree with those of van Agtmael et al. (37) that dihydroartemisinin paralleled the pharmacokinetics of artemether and reached a higher *C*_max_ and AUC_0–8_, as demonstrated in the plasma concentration-time profiles of artemether and dihydroartemisinin alone in unstratified volunteers and patients on nevirapine-based ART regimens, as presented in Fig. 3 .

The nonsignificantly lower exposure to artemether in the presence of nevirapine observed when stratified to *CYP2B6* c.516TT patients only can be attributed to influence of the mutant c.516T allele, which has been associated with decreased CYP2B6 expression (20) and thus decreased amount of CYP2B6 for nevirapine to induce and consequently increase artemether clearance. Only 23% higher artemether CL/F was observed in TT compared to the 137% seen in GG patients. For this reason, the observed reduction in artemether exposure was 62% greater in GG compared to TT subjects (Table S1). The innate and stable increase in the exposure to artemether due to decreased CYP2B6 expression and clearance in TT patients overwhelmed any nevirapine-related reduction in exposure as a consequence of CYP2B6 and 3A4 induction. Hence, the nonsignificantly lower total exposure to artemether observed in TT compared to the significantly lower exposure in GG patients is an indication of CYP2B6 involvement in artemether disposition. In addition, the higher exposure to nevirapine of TT compared to GG patients previously reported (36) may have contributed greatly to the significantly higher exposure to dihydroartemisinin due to the combined effect of CYP2B6 and 3A4 induction in the biotransformation of artemether.

Although mainly expressed in the liver, CYP3A4 in particular, and 2B6 to a lesser extent are also expressed in the small intestines (38). Consequently, artemether is also subjected to high intestinal biotransformation leading to low bioavailability, as well as increased susceptibility to other drug-drug interactions. Moreover, artemether autoinduction due to increased CYP2B6 activity (39) following repeated administration cannot be completely ruled out, further contributing to the reduction in artemether bioavailability. These, in addition to the influence of genetic polymorphisms in artemether-metabolizing enzymes and transport proteins, may account for the wide interindividual variations in artemether pharmacokinetics as observed in this study.

The present study also showed that lumefantrine and desbutyl-lumefantrine pharmacokinetic parameters of GG subjects in the artemether-lumefantrine alone subgroup were not too different from the parameters obtained in the same subgroup for all the unstratified subjects (e.g., a lumefantrine AUC_0–∞_ of 431,000 [123,000] versus 441,000 [138,000] ng ⋅ h/ml; *P* = 0.735). This is a clear demonstration of the noninvolvement of CYP2B6 in the lumefantrine to desbutyl-lumefantrine biotransformation. The observed differences in parameters in the artemether-lumefantrine plus nevirapine subgroup (e.g., lumefantrine AUC_0–∞_ of 466,000 [240,000] versus 573,000 [292,000] ng ⋅ h/ml; *P* = 0.259) were due to the inductive effect of nevirapine, which incidentally was also influenced by *CYP2B6* polymorphisms (14, 20, 36). The overall 30% higher exposure to lumefantrine when not stratified to *CYP2B6* c.516G>T genotypes is consistent with data from previous similar parallel design studies (29, 32–34). Kredo et al. (29) recruited ART-naive patients as control and reported 56% significantly higher lumefantrine exposure. Chijioke-Nwauche et al. (32) recruited HIV-negative subjects as controls and obtained 29% higher day 7 plasma lumefantrine levels versus the observed 37% rise in the present study’s day 6 plasma lumefantrine levels (*C*_day 6_). The Maganda et al. (33) study in HIV- and malaria-coinfected patients recruited ART naive patients as control and reported 25% nonsignificantly higher lumefantrine exposure compared to the 30% rise obtained in this study. A recent study in Ugandan and Malawian HIV-infected children have also reported 123% greater plasma exposure to lumefantrine of children on nevirapine-based ART using HIV-negative historical controls (34). However, the results of this study disagree with a similar parallel design study in HIV-infected Nigerian patients that reported 49% lower lumefantrine exposure using HIV-negative healthy volunteers as control (31). It is also not in agreement with the crossover design study in HIV-infected Ugandan patients that reported a 21% nonsignificant decrease (30).

While artemether and dihydroartemisinin rapidly clear most of the infection, lumefantrine concentrations that remain at the end of days 3 to 5 after the first dose are responsible for eliminating the residual parasites (40). Moreover, the day 7 plasma concentration has proved to be a good predictor of day 28 recurrence and an important determinant of the therapeutic response (41). Day 7 plasma lumefantrine concentration cutoff values of 175 and 280 ng/ml after the first dose have been reported (42–44). Although the study day 7 concentration after the first dose was not available for direct comparison, no patients were observed to have a day 6 plasma lumefantrine concentration below the cutoff value of 280 ng/ml. In addition, of the total 29 patients with day 10 lumefantrine concentrations (*C*_day 10_), 5 patients (i.e., approximately 19%) had concentrations below the cutoff value of 280 ng/ml, and all were found to be of GG genotype (as indicated in Fig. 2). The average CD4 count (standard deviation) for these five patients was 340 (300) cells/mm^3^, greater than the overall mean CD4 count of the GG patients (327 [207] cells/mm^3^). Thus, lower host immunity can be ruled out as being responsible for the observed reduction in the plasma lumefantrine concentrations of these patients below the cutoff value. The 24% significantly lower exposure to desbutyl-lumefantrine after nevirapine coadministration in this group of subjects compared to their TT counterparts (Table S2) may explain this finding. A previous *in vitro* study had shown desbutyl-lumefantrine to be more potent than lumefantrine and to play stronger role in the suppression of recrudescence, its relatively low plasma concentration notwithstanding (45).

The results of this study showed that nevirapine-based ART phenotypically reduced artemether exposure with a corresponding rise in dihydroartemisinin exposure, as against the genotype-predicted rise in artemether and the reduction in dihydroartemisinin exposures in the absence of nevirapine, in *CYP2B6* c.516TT subjects. In addition, although CYP2B6 is not the main enzyme responsible for the biotransformation of lumefantrine (10), the higher exposure to nevirapine of TT patients observed in our previously reported study (36) culminated in greater exposure to lumefantrine compared to their GG counterparts. Therefore, coadministration of nevirapine with artemether-lumefantrine favored higher exposures to the two principal determinants of antimalarial efficacy as dihydroartemisinin and lumefantrine exposures were approximately 4 and 46%, respectively, higher in TT than in GG patients (Tables 2 and 4). Generally, lower artemether-to-dihydroartemisinin and higher lumefantrine-to-desbutyl-lumefantrine metabolic ratios are expected to favor antimalarial efficacy, and both were significantly reduced and enhanced, respectively, in both genotype groups, although to a greater magnitude in TT than in GG patients. Data from this study, along with a previous study (46), demonstrate the impact of genetic polymorphisms on the severity and magnitude of drug-drug interactions. Thus, genotyping for polymorphic drug-metabolizing enzymes and transporters is a useful strategy to improve the prediction and interpretation of drug interaction outcomes. More studies of other genetically predicted drug-drug interactions are warranted to assess the impact of pharmacogenetics on the magnitude and severity of such interactions.

This was a small exploratory study with two comparisons of interest, i.e., the nevirapine effect (a within-group test for the presence versus the absence of nevirapine) and the genotype effect (a between-group test for *CYP2B6* c.516TT versus GG in the presence of nevirapine). Therefore, no adjustments were made for multiple comparisons. Rather, effect sizes and the associated confidence intervals and *P* values are presented to permit individual interpretation of relative weight of the results and conclusion. Comparison of genotype effect on artemether-lumefantrine pharmacokinetics in the absence of nevirapine has been described elsewhere (47). Furthermore, the relatively small sample sizes (particularly in the GG subgroups) may have reduced the power to detect differences in some of the pharmacokinetic parameters. A *post hoc* statistical power analysis conducted for the smallest sample sizes (*n* = 8 and *n* = 10 for both within-group and between-group tests) using G*Power (v3.9.1.4) indicated a 35.8% reduction in the power to detect differences compared to the achieved power of *n* = 15 in each of the subgroups for a two-tailed *t* test difference between two independent means using a medium effect size of 0.5.

In addition, this study investigated effect of homozygosity for *CYP2B6* c.516G>T without consideration for the influence of heterozygosity of this polymorphism. The frequency of GT genotype was 42% (28) in the sampled patients’ population, an indication of the fact that majority of the subjects were excluded. Moreover, *CYP2B6* c.983T>C (*18), which is similarly associated with significant reduction in CYP2B6 catalytic activity (48) and also common in African population (49), though with a minor allele frequency of <10% compared to <50% of the c.516G>T, was also not factored into the study due to the limited funds available. Inclusion of c.983T>C would have compensated for the relatively small sample size and improved the power to detect differences in the parameters since heterozygous carriers of both c.516T and c.983C alleles would have been classified as slow metabolizers, adding more power to the study. Therefore, subsequent larger definitive studies with adequate power and based on composite *CYP2B6* c.516/983 genotype are needed to confirm the observed associations.

Other potential limitations of this study include the inability to recruit sex-matched retroviral negative volunteers as controls. In addition, differences in participant disease state and immune status (HIV-infected patients versus HIV-negative controls) may have also contributed to the observed interindividual variabilities in artemether-lumefantrine pharmacokinetics. A previous study has demonstrated that HIV-infected patients had 18% lower hepatic CYP3A4 activity, as measured by midazolam plasma clearance compared to age- and sex-matched healthy volunteers (50). Although it is not clear whether HIV infection also has an impact on CYP2B6 activity, HIV infection is generally associated with an increase in the variability of drug-metabolizing enzymes. Furthermore, nonadherence cannot be absolutely excluded since sampled participants self-administered the first five doses of artemether-lumefantrine at home, and only their last doses were observed in the clinic. Despite these limitations, the study results compare favorably with previous studies that recruited ART-naive HIV-infected patients as controls, as well as studies involving HIV-infected patients with clinical malaria (29, 33). However, more crossover designed studies are required to substantiate the decreased lumefantrine exposure as a result of nevirapine coadministration reported by Byakika-Kibwika et al. (30).

In summary, data from this study underline the importance of the *CYP2B6* c.516G>T genotype in the pharmacokinetic interactions of nevirapine with artemether-lumefantrine. Although nevirapine only reduced exposures to artemether and desbutyl-lumefantrine in GG subjects, it enhanced exposures to dihydroartemisinin and lumefantrine in TT subjects on nevirapine-based ART. Incorporation of pharmacogenetics in all relevant drug-drug interaction studies is necessary for a better understanding and interpretation of data.

## MATERIALS AND METHODS

### Study population and design.

This was a prospective, open-label, two-arm parallel drug-drug interaction study in HIV-negative volunteers (*n* = 30) and HIV-infected patients (*n* = 30) drawn from cohorts of 150 participants each, with previously determined *CYP2B6* c.516G>T genotypes (28). Both the HIV-negative and HIV-infected subjects were without clinical malaria. Patients were eligible for the study if they were at least 18 years old; homozygous wild type, i.e., noncarrier or homozygous carrier of the variant T allele; receiving ART containing nevirapine for at least 2 months; and had no recent history of poor adherence. Individuals were excluded if they had taken artemether-lumefantrine within the previous ≤30 days of pharmacokinetic sampling, were allergic to artemether or lumefantrine, were pregnant, were breastfeeding, used substances or drugs with known or unknown interaction with artemether or lumefantrine, were patients on ritonavir-boosted lopinavir, or had history of acute or chronic illnesses. Written informed consent was obtained from all participants prior to enrollment and OAUTHC Health Research Ethics Committee (HREC) granted ethics approval for the study (ERC/2013/06/01) and Materials Transfer Agreement was approved by the National Health Research Ethics Committee, Abuja, Nigeria (NHREC/01/01/2007-02/05/204).

### Drug administration and pharmacokinetic sampling for artemether and lumefantrine.

HIV-infected participants were sent regular reminders to enhance adherence to their ART during the 2 weeks preceding artemether-lumefantrine administration. After we confirmed ongoing eligibility, participants in both groups were given 24 tablets of coformulated 20 mg of artemether and 120 mg of lumefantrine. They were instructed to self-administer four tablets each after a standardized meal at 12 midnight on day 1, again 8 h later, and then every 12 h in line with the prescribing information. Apart from the first dose, HIV-infected patients took subsequent artemether-lumefantrine doses within a few minutes of taking their ART. The sixth artemether-lumefantrine dose was directly observed by study staff at 8 a.m. on day 3 before intensive pharmacokinetic sampling. Blood samples (ca. 4 ml) were collected in lithium-heparinized plasma separating tubes just before and 0.5, 1, 2, 3, 4, 6, 8 (day 3), 24 (day 4), 72 (day 6), 96 (day 7), 168 (day 10), and 336 h after the sixth artemether-lumefantrine dose. Plasma was separated from whole blood by spinning at 3,000 × *g* for 10 min and stored at –80°C in cryovials until analysis. Plasma samples were shipped on dry ice to Mahidol-Oxford Tropical Medicine Research Unit (MORU) Clinical Pharmacology Laboratory, Bangkok, Thailand, for the assay of artemether and lumefantrine.

### Liquid chromatography-tandem mass spectrometry assays and pharmacokinetic analysis.

Quantification of artemether, dihydroartemisinin, lumefantrine, and desbutyl-lumefantrine in plasma samples was performed using previously validated liquid chromatographic-tandem mass spectrometric methods (51–53). Briefly, the lower limits of quantification were 1.43 ng/ml for artemether and dihydroartemisinin, 9.71 ng/ml for lumefantrine, and 1.01 ng/ml for desbutyl-lumefantrine. The coefficients of variation were lower than 4.0% for both artemether and dihydroartemisinin and 6.5% for lumefantrine and desbutyl-lumefantrine for all the quality control samples.

Noncompartmental analysis of plasma drug concentrations of artemether, dihydroartemisinin, lumefantrine, and desbutyl-lumefantrine was performed using Kinetica (v4.1; InnaPhase Corp., Philadelphia, PA). The following pharmacokinetic parameters were calculated for all drugs: observed peak plasma concentration (*C*_max_), time to reach *C*_max_ (*T*_max_), area under the concentration-time curve from time zero to the last (AUC_0–_*_t_*), total AUC from time zero to infinity (AUC_0–∞_), half-life of the terminal elimination phase (*t*_1/2_), oral clearance (CL/F), and the metabolic ratio of the parent drug’s AUC_0–∞_ to the metabolite’s AUC_0–∞_. Artemether and lumefantrine were assumed to be fully transformed into dihydroartemisinin and desbutyl-lumefantrine *in vivo* and the relative difference in molecular weights (i.e., dihydroartemisinin/artemether or desbutyl-lumefantrine/lumefantrine) were used to calculate the putative doses of administered dihydroartemisinin (76.24 mg) and desbutyl-lumefantrine (429.08 mg) (54).

### Statistical analysis.

Pharmacokinetic parameters are presented as geometric means (standard deviations) except for *T*_max_, which is presented as median (interquartile range), to three significant figures, stratified on HIV and genotype status. The ratio of the geometric mean (RoGM) and the associated 90% confidence interval (CI) were used to assess the magnitude of change for each of the pharmacokinetic parameters. RoGMs were obtained by dividing geometric means of the artemether-lumefantrine pharmacokinetic parameters when coadministered with nevirapine by geometric means of the parameters alone for the within-group comparison. The impact of nevirapine coadministration was also stratified on *CYP2B6* c.516GG and TT genotypes to assess the impact of genotype on the drug-drug interaction. The RoGMs for this between-group comparison were obtained by dividing geometric means of the artemether-lumefantrine plus nevirapine parameters of TT by GG subjects. The results were considered clinically significant if the 90% CI did not fall entirely within the 0.80 to 1.25 no-effect boundary in accordance with U.S. Food and Drug Administration guidelines (55). All statistical analyses were performed using SPSS statistics (v20.0; IBM, Armonk, NY), and figures were produced using Prism version 6.01 (GraphPad Software, Inc., San Diego, CA).

### Data availability.

Data for artemether, lumefantrine, and the *CYP2B6* c.516G>T genotype can be found in the Dryad database (doi: 10.5061/dryad.z612jm683) (56).

## Supplementary Material

Supplemental file 1

Supplemental file 2
